# Dynamic conformal arcs for lung stereotactic body radiation therapy: A comparison with volumetric‐modulated arc therapy

**DOI:** 10.1002/acm2.12800

**Published:** 2019-12-27

**Authors:** Rasmus Bokrantz, Minna Wedenberg, Peter Sandwall

**Affiliations:** ^1^ RaySearch Laboratories Stockholm Sweden; ^2^ Department of Radiation Oncology OhioHealth Mansfield OH USA

**Keywords:** conformal arcs, DCA, lung SBRT, VMAT

## Abstract

This study constitutes a feasibility assessment of dynamic conformal arc (DCA) therapy as an alternative to volumetric‐modulated arc therapy (VMAT) for stereotactic body radiation therapy (SBRT) of lung cancer. The rationale for DCA is lower geometric complexity and hence reduced risk for interplay errors induced by respiratory motion. Forward planned DCA and inverse planned DCA based on segment‐weight optimization were compared to VMAT for single arc treatments of five lung patients. Analysis of dose‐volume histograms and clinical goal fulfillment revealed that DCA can generate satisfactory and near equivalent dosimetric quality to VMAT, except for complex tumor geometries. Segment‐weight optimized DCA provided spatial dose distributions qualitatively similar to those for VMAT. Our results show that DCA, and particularly segment‐weight optimized DCA, may be an attractive alternative to VMAT for lung SBRT treatments if the patient anatomy is favorable.

## 1 INTRODUCTION

Stereotactic body radiation therapy (SBRT) is standard of care for inoperable non‐small‐cell lung cancer (NSCLC).^1^ In the United States, SBRT for NSCLC is delivered in 1–5 fractions and with up to 10 fractions internationally, both using biological effective doses in excess of 100 Gy.^2^ Radiation dose delivery to moving targets, such as lung tumors, has been a fundamental challenge in radiation oncology. Traditional approaches to account for motion have entailed expansion of the gross or clinical target volume to include the entire range of motion; defined as the internal target volume (ITV).^3^ Several other devices and strategies have been developed to manage and minimize the effects of respiratory motion, including compression and breath hold devices.^4^ A recent advance is the development of robust radiotherapy plans, where the uncertainty in the target location is parameterized in the optimization.^5^

Currently, the most common approach for lung SBRT is treatment to an ITV using volumetric‐modulated arc therapy (VMAT), where dose is delivered during an arc with simultaneous dynamic motion of the multi‐leaf collimator (MLC) leaves.^6^ Current optimization methods do not constrain leaf motion to prevent occlusion of the target. This form of treatment delivery is susceptible to dosimetric errors from unexpected interplay between organ motion and MLC leaf motion — a phenomenon termed the interplay effect.^7^, ^8^ The interplay effect can create significant dosimetric deviations greater than 20%; however, the effect averages out over traditionally fractionated (>25) courses of intensity‐modulated radiation therapy (IMRT).^9^ Studies on lung SBRT have shown the necessity of using multiple arcs to obtain the averaging benefit for hypofractionated VMAT.^10^, ^11^

**Figure 1:**
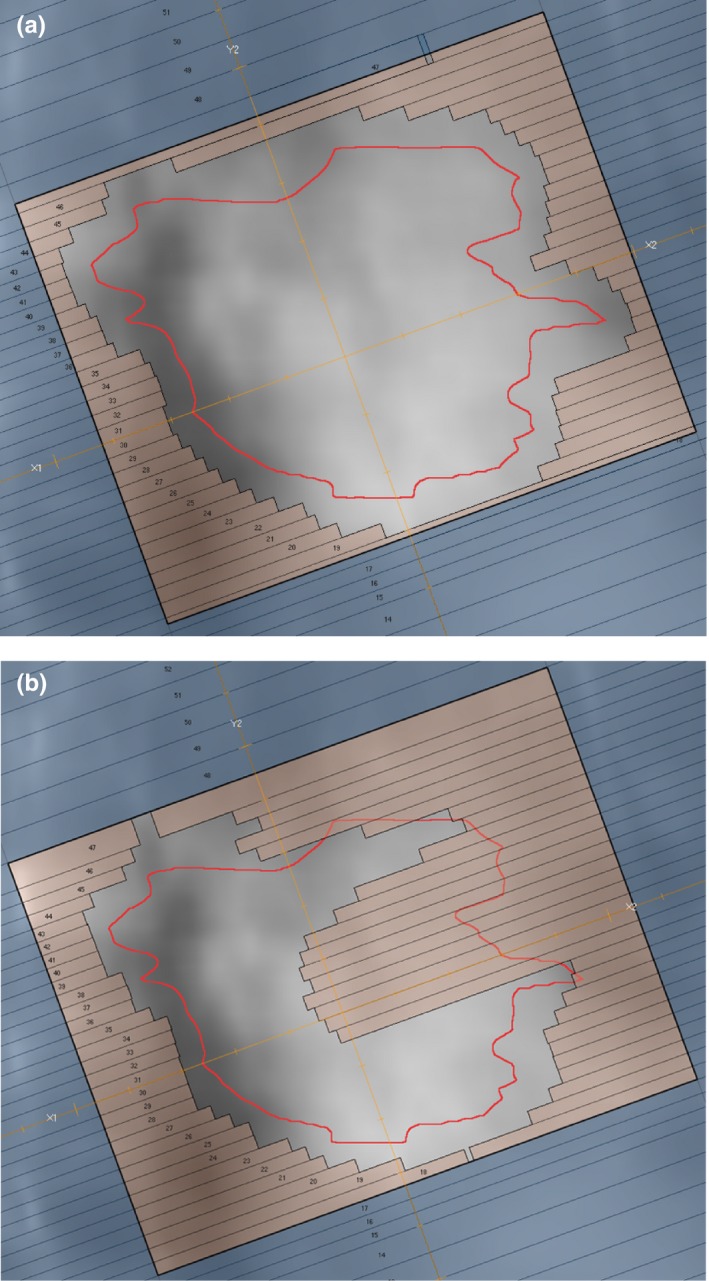
Typical segment shapes for (a) dynamic conformal arc (DCA) therapy and (b) volumetric‐modulated arc therapy (VMAT) treating a target volume indicated by the red contour. Multi‐leaf collimator (MLC) leaves are shown in brown and jaws shown in blue.

Dynamic conformal arc (DCA) treatments generated by forward planning are known to be efficient and clinically effective for lung SBRT as they eliminate or reduce concerns of the interplay effect.^12^, ^13^, ^14^ The contrast between DCA and VMAT is illustrated in Fig. 1. Note that the VMAT plan in this figure has some MLC leaves occluding the target, whereas the leaves are conformed to the shape of the target for the DCA plan. Dosimetric accuracy has also been demonstrated to decrease with increased modulation complexity and increased leaf travel.^15^ With the high degree of precision required for hypofractionated treatments, it is important to ensure radiotherapy plans are adequately but not overly complex.^16^, ^17^

This study reviews the DCA planning method within the RayStation (RaySearch Laboratories, Stockholm, Sweden) treatment planning system and compares DCA to VMAT plans, highlighting the appropriate selection of each technique. We also evaluate this system's capability to generate segment‐weight optimized dynamic conformal arc (SWO‐DCA) plans, where a nonuniform number of monitor units (MUs) as a function of the gantry angle is determined by inverse planning techniques. Note that treatment planning for DCA is supported also in other treatment planning systems, such as Eclipse (Varian Medical Systems, Palo Alto, CA) and Monaco (Elekta, Stockholm, Sweden).

RayStation's workflow for each plan generation technique is described in greater detail below. A common prerequisite is that the basic geometric properties of the arcs have been defined, such as isocenter position, start and stop gantry angle, collimator and couch angle, and gantry angle spacing between control points.

- DCA: Target volumes for each arc and a margin for each combination of arc and target are first selected. Also, a leaf positioning strategy is selected that determines how the MLC leaves are aligned with the boundary of the beam's‐eye‐view projection of the target. Leaves can either be positioned to not overlap with the target, be positioned so that no nontarget area is exposed between the leaves and the target or be placed at intermediate positions relative to the other two strategies. The system conforms the MLC leaves while accounting for dynamic motion constraints such as the maximum MLC leaf speed and the maximum gantry angle speed, as necessary since the shape of the conformed MLC changes as function of the gantry angle. A uniform scaling of the number of MUs per arc is finally performed toward fulfillment of a prescription criterion, for example, that the average dose or a dose‐at‐volume level for a target should equal a selected dose level.
- VMAT: An optimization problem is defined in terms of objective functions and constraints assigned to the targets and organs at risk (OARs). The system generates a treatment plan by optimizing the MLC leaf positions and number of MUs of each control point of the arcs. RayStation's algorithm for VMAT optimization is described in detail in Bzdusek et al.^18^
- SWO‐DCA: A standard DCA plan is first created, as per above. An optimization problem is then defined similar to VMAT optimization and the number of MUs per control point of the arcs optimized according to this definition. The MLC leaf positions are kept unchanged during the optimization process.

## 2 METHODS

Treatment plans were generated for five anonymized patients (patients 1–5) using RayStation 7.0. A subset of the patient cases was obtained from the 4D lung collection of The Cancer Imaging Archive.^19^ Treatment planning was performed with respect to the average image derived from all phases of a four‐dimensional computed tomography (4DCT) dataset. An ITV was created using the maximum intensity projection 4DCT. All patients were planned for treatment with a single coplanar arc with a 6 MV energy beam from a TrueBeam linear accelerator (Varian Medical Systems, Palo Alto, CA), with isocenter position set to the center of the ITV. Individual arc lengths in the range 210–240 degrees and collimator angles in the range 0–20 degrees where selected for each patient case. These angles were selected based on which values that were found suitable with respect to fulfillment of clinical goals. No couch rotations were used. The fractionation schedule used was 60 Gy in 8 fractions. One treatment plan per evaluated plan generation technique were created for each patient. The DCA plans were created by conforming the MLC to the ITV with an isotropic margin of 0.5 cm according to the leaf positioning strategy where no overlap with the expanded target occurs. The number of MUs of the arc was the scaled to achieve D95% to the prescription level, whereas the SWO‐DCA and VMAT plans were generated by optimization with respect to objectives defined in accordance with patient‐specific clinical goals, as summarized in Tables 2−3.

Treatment plans were evaluated with respect to level of clinical goal fulfillment and dose‐volume histograms (DVHs). Treatment plan complexity was assessed in term of the modulation complexity score (MCS), the score being introduced by McNiven, et al.^20^ for static‐field IMRT and later adapted to VMAT by Masi, et al.^15^ The MCS score depends on the leaf position variability between adjacent active leaves and the aperture area variability. The score is dimensionless and ranges from 0 to 1, with a value of 1 corresponding to the lowest possible complexity (a rectangular field). The set of active leaf pairs used for the MCS evaluation was defined as the leaf pairs with a tip gap inside the jaw opening that is greater than the minimum dynamic tip for at least one control point. Plan complexity was also assessed in terms of total leaf travel, averaged over the active leaf pairs, and total MU variability.

## 3 RESULTS

**Figure 2:**
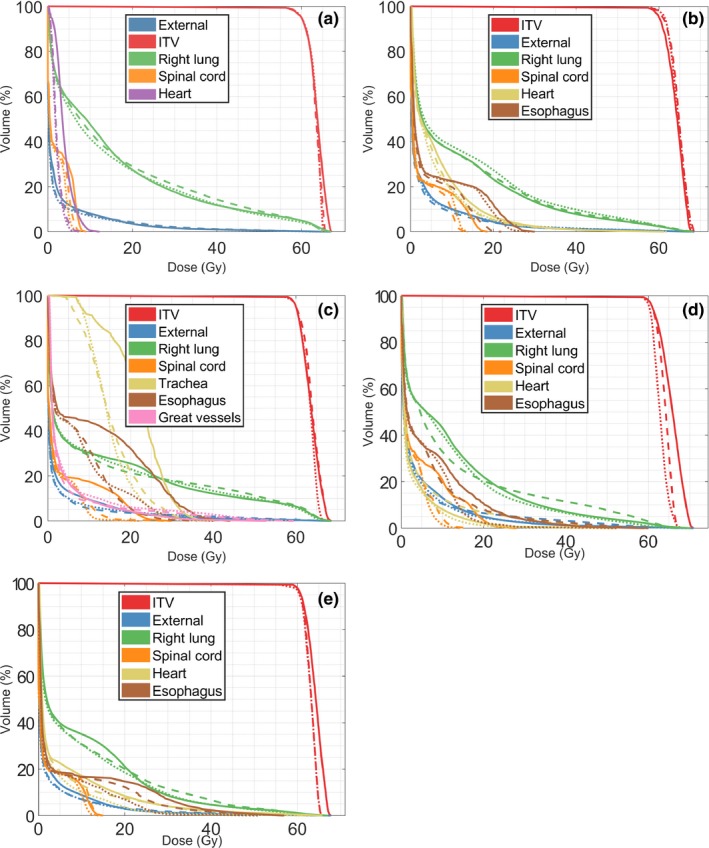
Dose‐volume histograms (DVHs) for the five patient cases. The standard dynamic conformal arc (DCA) plan is indicated by solid lines, the segment‐weight optimized (SWO)‐DCA plan indicated by dashed lines, and the volumetric‐modulated arc therapy (VMAT) plan indicated by dotted lines. (a) Patient 1, (b) Patient 2, (c) Patient 3, (d) Patient 4, (e) Patient 5.

**Figure 3:**
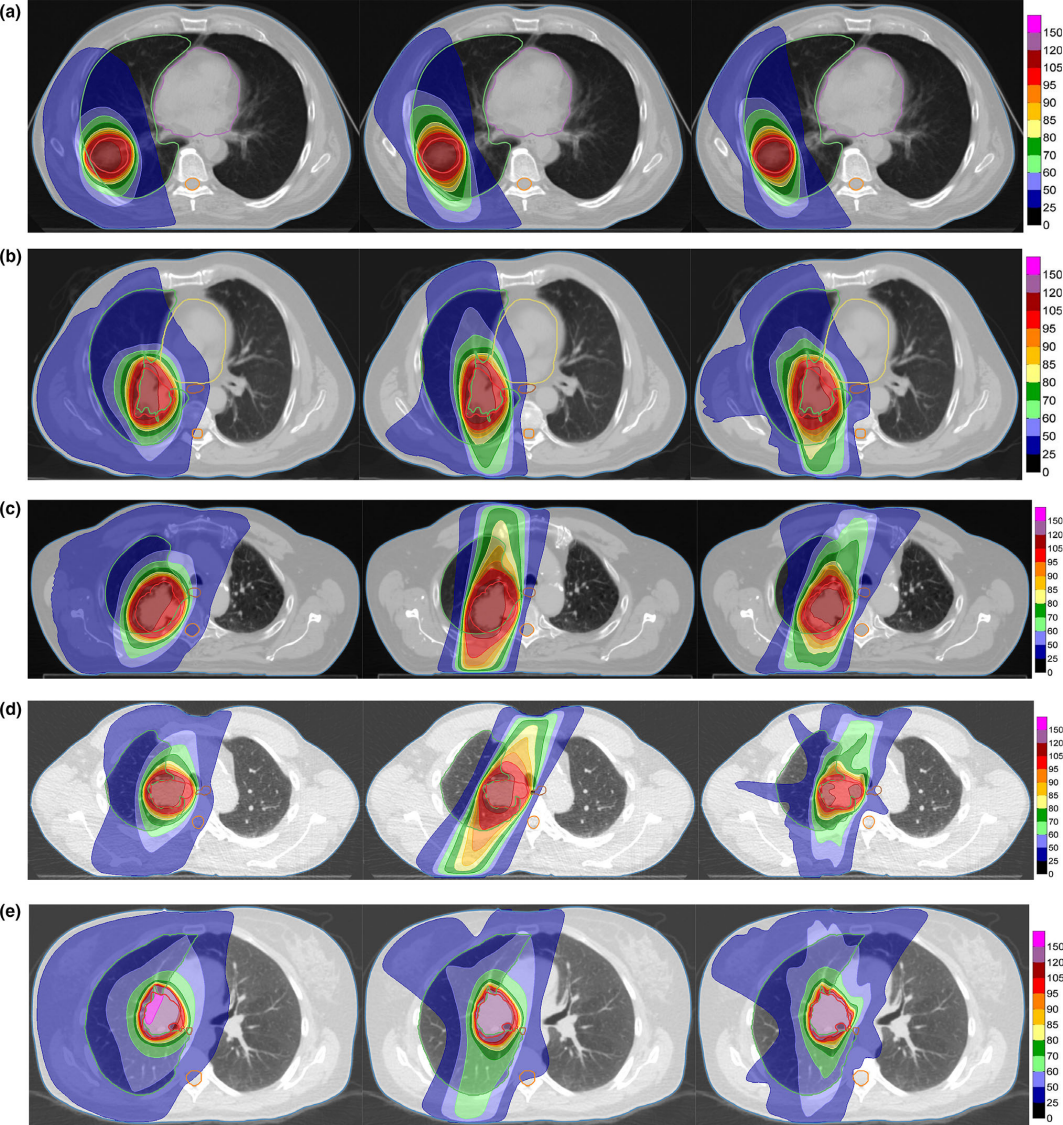
Dose distributions for the five patient cases for a transversal cut through the isocenter, overlaid on the planning computed tomography (CT). The plans shown in each subfigure are (from left to right): dynamic conformal arc (DCA), segment‐weight optimized (SWO)‐DCA, and volumetric‐modulated arc therapy (VMAT). Contours for regions of interests (ROIs) are indicated by solid lines according to the color scheme of Fig. 2. The color table is in percent of the prescription dose (60 Gy). (a) Patient 1, (b) Patient 2, (c) Patient 3, (d) Patient 4, (e) Patient 5.

The dose distributions of the treatment plans are illustrated with DVHs in Fig. 2 and 2D dose distributions for a transversal slice shown in Fig. 3. The contours for regions of interest (ROIs) are here indicated in colors in accordance with Fig. 2. The examined plan complexity metrics are summarized in Table 1 and the level of clinical goal fulfillment per patient case summarized in Tables 1−2.

**Table 1:** Summary of obtained plan complexity metrics per patient case and plan generation technique.

Case	Metric	DCA	SWO‐DCA	VMAT
Patient 1	MCS (−)	0.62	0.63	0.60
	LT (cm)	2.5	2.5	4.6
	∆MU (MU)	0.0	25.5	34.9
Patient 2	MCS (−)	0.45	0.43	0.45
	LT (cm)	5.1	5.1	14.6
	∆MU (MU)	0.0	60.9	138.0
Patient 3	MCS (−)	0.51	0.50	0.37
	LT (cm)	2.9	2.9	21.0
	∆MU (MU)	0.0	103.3	129.1
Patient 4	MCS (−)	0.41	0.42	0.26
	LT (cm)	2.4	2.4	21.4
	∆MU (MU)	0.0	124.2	181.6
Patient 5	MCS (−)	0.47	0.46	0.28
	LT (cm)	3.2	3.2	20.0
	∆MU (MU)	0.0	94.3	174.0

∆MU, total MU variation; DCA, dynamic conformal arc; LT, total leaf travel; MCS, modulation complexity score; SWO, segment‐weight optimized; VMAT, volumetric‐modulated arc therapy.

**Table 2:** Fulfillment of clinical goals on maximum dose at volume for Patient 1.

ROI	Maximum dose at volume	Resulting dose (Gy) at volume		
		DCA	SWO‐DCA	VMAT
Spinal cord	13.5 Gy at 0.5 cm^3^	7.93	6.96	6.16
	22.5 Gy at 0.25 cm^3^	8.07	7.19	6.43
	30.0 Gy at 0 cm^3^	9.01	8.19	7.40
Lungs	12.5 Gy at 1500 cm^3^	2.71	1.78	1.59
	13.5 Gy at 1000 cm^3^	8.74	7.40	6.36
Heart	32.0 Gy at 15 cm^3^	8.87	5.45	4.90
	63.0 Gy at 0 cm^3^	12.2	8.39	7.17
Trachea	18.0 Gy at 4 cm^3^	1.25	0.87	0.80
	63.0 Gy at 0 cm^3^	12.0	6.89	6.48

Satisfied goals are indicated in green.

DCA, dynamic conformal arc; ROI, region of interest; SWO, segment‐weight optimized; VMAT, volumetric‐modulated arc therapy.

**Table 3:** Fulfillment of clinical goals on maximum dose at volume for Patient 2.

ROI	Maximum dose at volume	Resulting dose (Gy) at volume		
		DCA	SWO‐DCA	VMAT
Spinal cord	13.5 Gy at 0.5 cm^3^	16.7	11.7	12.6
	22.5 Gy at 0.25 cm^3^	17.0	12.4	13.1
	30.0 Gy at 0 cm^3^	18.6	13.3	13.9
Lungs	12.5 Gy at 1500 cm^3^	5.53	4.16	5.31
	13.5 Gy at 1000 cm^3^	9.07	7.56	9.84
Heart	32.0 Gy at 15 cm^3^	30.0	28.5	29.0
	63.0 Gy at 0 cm^3^	61.3	61.3	62.3
Esophagus	27.5 Gy at 5 cm^3^	20.8	14.4	19.4
	63.0 Gy at 0 cm^3^	30.0	21.9	28.7

Satisfied goals are indicated in green and violated goals indicated in red.

DCA, dynamic conformal arc; ROI, region of interest; SWO, segment‐weight optimized; VMAT, volumetric‐modulated arc therapy.

**Table 4:** Fulfillment of clinical goals on maximum dose at volume for Patient 3.

ROI	Maximum dose at volume	Resulting dose (Gy) at volume		
		DCA	SWO‐DCA	VMAT
Spinal cord	13.5 Gy at 0.5 cm^3^	25.0	17.2	12.7
	22.5 Gy at 0.25 cm^3^	25.9	18.8	14.6
	30.0 Gy at 0 cm^3^	30.0	29.8	22.5
Lungs	12.5 Gy at 1500 cm^3^	11.3	0.89	0.96
	13.5 Gy at 1000 cm^3^	44.3	2.31	2.46
Heart	32.0 Gy at 15 cm^3^	0.65	0.61	0.65
	63.0 Gy at 0 cm^3^	0.87	0.78	0.84
Trachea	18.0 Gy at 4 cm^3^	25.8	17.9	16.5
	63.0 Gy at 0 cm^3^	40.2	40.3	38.6
Esophagus	27.5 Gy at 5 cm^3^	24.6	12.5	11.5
	63.0 Gy at 0 cm^3^	44.1	36.0	40.0
Great vessels	47.0 Gy at 10 cm^3^	17.2	19.5	25.2
	63.0 Gy at 0 cm^3^	52.7	59.0	59.9

Satisfied goals are indicated in green and violated goals indicated in red.

DCA, dynamic conformal arc; ROI, region of interest; SWO, segment‐weight optimized; VMAT, volumetric‐modulated arc therapy.

**Table 5:** Fulfillment of clinical goals on maximum dose at volume for Patient 4.

ROI	Maximum dose at volume	Resulting dose (Gy) at volume		
		DCA	SWO‐DCA	VMAT
Spinal cord	13.5 Gy at 0.5 cm^3^	23.1	12.7	11.1
	22.5 Gy at 0.25 cm^3^	24.2	13.6	12.0
	30.0 Gy at 0 cm^3^	28.0	16.7	14.4
Lungs	12.5 Gy at 1500 cm^3^	3.8	2.6	3.6
	13.5 Gy at 1000 cm^3^	7.0	5.1	6.6
Heart	32.0 Gy at 15 cm^3^	5.6	8.1	5.2
	63.0 Gy at 0 cm^3^	52.2	58.3	44.5
Esophagus	27.5 Gy at 5 cm^3^	26.5	26.9	16.3
	63.0 Gy at 0 cm^3^	58.9	60.0	52.2

Satisfied goals are indicated in green and violated goals indicated in red.

DCA, dynamic conformal arc; ROI, region of interest; SWO, segment‐weight optimized; VMAT, volumetric‐modulated arc therapy.

**Table 6:** Fulfillment of clinical goals on maximum dose at volume for Patient 5.

ROI	Maximum dose at volume	Resulting dose (Gy) at volume		
		DCA	SWO‐DCA	VMAT
Spinal cord	13.5 Gy at 0.5 cm^3^	13.5	13.5	12.1
	22.5 Gy at 0.25 cm^3^	13.9	13.7	12.4
	30.0 Gy at 0 cm^3^	15.0	14.6	13.9
Lungs	12.5 Gy at 1500 cm^3^	4.6	2.7	2.4
	13.5 Gy at 1000 cm^3^	7.7	5.8	5.9
Heart	32.0 Gy at 15 cm^3^	35.2	33.8	21.4
	63.0 Gy at 0 cm^3^	65.8	62.3	63.0
Esophagus	27.5 Gy at 5 cm^3^	33.2	27.5	22.1
	63.0 Gy at 0 cm^3^	56.7	57.4	50.8

Satisfied goals are indicated in green and violated goals indicated in red.

DCA, dynamic conformal arc; ROI, region of interest; SWO, segment‐weight optimized; VMAT, volumetric‐modulated arc therapy.

For patient 1, all clinical goals were achieved with the three treatment techniques (Table 2). For patient 2, clinical goals were achieved, with exception of the spinal cord with the standard DCA plan (Table 3). Patient 3 had a more complex geometry, with a large centrally located target proximal the spinal cord. In this case, neither DCA nor SWO‐DCA plans could be created to fulfill all clinical goals (Table 4). The standard DCA plan failed on sparing of the spinal cord and trachea, whereas the SWO‐DCA plan failed the spinal cord. In addition to improved sparing of the trachea, SWO‐DCA also led to improved sparing of the esophagus compared to standard DCA, as evident in Fig. 2(c). The VMAT plan fulfilled all goals for patient 3 (Table 4). Results for patient 4 were similar to those for patient 2: clinical goals were achieved except for goals on sparing of the spinal cord with the standard DCA plan (Table 5). Patient 5 had a challenging geometry with the target located in direct vicinity of both the heart and esophagus. The standard DCA plan violated a total of three goals on sparing of these OARs, whereas the SWO‐DCA plan violated a single goal on sparing of the heart. The VMAT plan fulfilled all goals for patient 5 (Table 6).

Figures 3 shows that standard DCA plans produce relatively symmetrical dose distributions. The SWO‐DCA and VMAT plans, in contrast, yielded heavily weighted anterior and posterior dose delivery for all five cases. The VMAT plans generally exhibited a higher level of complexity than standard DCA and SWO‐DCA plans according to evaluated complexity metrics. The exception to this general pattern was the MCS value for patient 2, which was lower for SWO‐DCA than VMAT. The lower MCS value (higher complexity) of the SWO‐DCA plan was due to a higher level of aperture area variability, which for this patient was caused by irregular target geometry. The VMAT plan is, arguably, the more complex plan having a factor 2.8 higher total leaf travel per degree and a factor 2.3 higher total MU variability.

## 4 DISCUSSION

Our results demonstrate DCA plans can achieve satisfactory and nearly equivalent plans to VMAT under favorable conditions. Based on the cases examined in this study, situations when DCA can provide dose distributions of comparable quality to VMAT are lesions that are not proximal to dose‐limiting OARs. We observed that complex cases with proximal OARs are better served with advanced treatment techniques such as VMAT. Nevertheless, our results show that SWO‐DCA provides a considerable benefit compared to standard DCA for complex cases, with treatment plans that are very close to fulfillment of all clinical goals. More large‐scale studies are warranted to establish if and under which circumstances that DCA is a favorable alternative to VMAT.

It was observed that segment weight optimization can considerably improve DCA plan quality with negligible change in plan complexity. Dose distributions of the SWO‐DCA plans were also observed to be similar to VMAT plans. Thus, we have demonstrated that DCA, and particularly SWO‐DCA, is a simple technique to create single arc lung SBRT plans of comparable quality with VMAT, while eliminating concerns of interplay and reducing complexity.

## CONFLICT OF INTEREST

This study was performed using the treatment planning system RayStation, manufactured by RaySearch Laboratories. The first and the second author are employees of RaySearch Laboratories.
